# Management of acute appendicitis during the COVID-19 pandemic: a retrospective cohort study

**DOI:** 10.1186/s12893-022-01851-1

**Published:** 2022-11-17

**Authors:** D. Frankcombe, N. Gauri, V. Satchithanandha, Y. Liang, S. Bak, T. Suri, D. Loxley, N. Merrett, D. Kaushal

**Affiliations:** 1 Department of Surgery, Campbelltown Public Hospital, Therry Road, Campbelltown, NSW 2560 Australia; 2 Department of Surgery, Royal Prince Alfred Hospital, Camperdown, Australia; 3 University of Western Sydney, Campbelltown, Australia

**Keywords:** Appendicitis, COVID-19, Pandemic, Emergency surgery

## Abstract

**Background:**

The Coronavirus Disease 2019 (COVID-19) pandemic profoundly impacted delivery of health care. South Western Sydney Local Health District (SWSLHD) experienced some of the highest cases, admissions and deaths during the Delta and Omicron waves in New South Wales. This study aims to determine the impact of the pandemic on emergency surgery services for adults presenting with acute appendicitis.

**Methods:**

A retrospective review of patient records was performed of adults presenting with acute appendicitis between 1st March 2021 and 31st March 2022, which was compared to a pre-COVID control period of the same dates in 2019–2020. Patients managed operatively or conservatively were included.

**Results:**

1556 patients were included in the operative arm; 723 and 833 respectively in the study and control groups, which were comparable at baseline. 1.66% were COVID positive. During the pandemic, patients were significantly more likely to be investigated with computered tomography (CT) scan (p ≤ 0.001), present with complicated appendicitis (p = 0.03), and require caecectomy (p = 0.005). They had higher American Society of Anaesthesiology (ASA) scores (p = 0.001) and significantly lower negative appendectomy rates (p = 0.001). Fifty-two patients were included in the conservative arm; 29 and 23 respectively in the pandemic and control groups. Patients were comparable at baseline. There were two COVID positive patients. During the pandemic, there was a significant reduction in complications (p = 0.033), readmissions (0.044) and interval appendicectomy (p = 0.0044).

**Conclusion:**

We identified higher rates of complicated appendicitis, caecectomies and greater reliance on CT imaging preoperatively during the pandemic in SWSLHD.

## Introduction

The Coronavirus Disease 2019 (COVID-19) pandemic, caused by severe acute respiratory syndrome coronavirus 2 (SARS-CoV2), has profoundly impacted the provision of health care. The virus and disease was first detected in December 2019 in Wuhan, China [1]. It was first recorded in Australia in a travellers returning to Sydney and Melbourne from Wuhan, China on the 25th of January 2020 [2]. To date, Australia has recorded in excess of 10 million cases and almost 15 thousand deaths [3]. The pandemic resulted in greater demand on hospital resources; including beds, staff and personal protective equipment, due to the many patients with COVID-19 requiring hospital admission [4]. The COVID pandemic also impacted supply due to supply-chain-issues, and staff absence due to illness or isolation.

The demand the pandemic had on Australian hospital resources was significant. During the first year of the pandemic, Australian emergency departments experienced a decrease in presentations by 1.4%, however this increased by 6.9% in the second year [5]. Across Australia there was a variation in the impact of the pandemic on emergency department presentations based on pathology, number of COVID-19 cases and geographic location [6–9]. Patient days in both public and private hospitals grew by 3.1% in 2021, an increase on the previous four year average of 0.2% [10]. In order to adapt to the increased demand, health care providers were required to alter their practice to ensure efficient use of hospital resources.

The South Western Sydney Local Health District (SWSLHD) is located in New South Wales (NSW), and services a population of more than 1 million people, accounting for 12.5% of the state population [11]. The area was significantly impacted during the Delta and Omicron waves of COVID 19, recording some of the highest case numbers, hospitalisations and intensive care admissions in the state of NSW [12, 13]. Furthermore, staff fourloughing due to illness and isolation requirements resulted in greater pressure on healthcare resources [14, 15]. As such, surgical departments servicing the district were required to promptly and efficiently assess and manage patients presenting with acute abdominal pain, in order to conserve resources.

Acute appendicitis is the most common cause for patients to present to the emergency department with abdominal pain, with approximately a 7% lifetime risk of occurrence [16]. It has a slight preponderance for males [17, 18]. Appendicitis is most commonly managed with appendicectomy, and it is the most commonly performed emergency operation in Australia [19]. As such, acute appendicitis is a good measure of the impact of the COVID-19 pandemic on the delivery of healthcare.

The aim of this study is to determine the impact the COVID-19 pandemic had on the provision of adult emergency surgery services; in particular patients presenting with acute appendicitis during the Delta and Omicron waves of 2021–22. We compared the rate of complications, severity of appendicitis, rates of conservative management, and a number of other outcomes in patients presenting during the COVID-19 pandemic and pre-COVID-19 pandemic to the three major hospitals servicing the SWSLHD.

## Methods

A retrospective study was conducted of adult admissions for acute appendicitis at three hospitals in the South Western Sydney Local Health District (Liverpool, Bankstown-Lidcombe and Campbelltown Hospitals). The study period was from 1 March 2021 to 31 March 2022 to cover the peak in cases associated with the Delta and Omicron variants of the COVID-19 pandemic. We compared this to the control period which was the same dates in 2019–2020. Human Research Ethics Committee approval was obtained for all sites (SWSLHD Ethics Committee 2022/ETH01147).

Patients were identified via their admission code (International Statistical Classification of Diseases and Related Health Problems, 10th revision, Australian Modification) codes K35-38 were used. Patients were included if they (1) were 18 years or older, (2) underwent an operation for presumed appendicitis, or were diagnosed with appendicitis radiologically and conservatively managed. Patients were excluded if (1) upon review of their record, the admission was not related to acute appendicitis, (2) alternative pathology was identified on laparoscopy and appendicectomy was not performed (e.g. ovarian torsion).

Conservative management was selected in patient’s presenting with phlegmonous appendicitis, in patients whom were medically unfit for operative management, or due to patient choice.

Data was extracted from the patient electronic medical record. This included patient demographics, COVID swab result, symptomatic COVID, patient reported duration of symptoms, method of diagnosis [clinical, computerised tomography (CT) or ultrasound (US)], operation performed, duration of operation, length of stay, time from admission to operation.

COVID swabs were routinely collected on all patients admitted to the hospital in the local health district from the 12th of July 2021. Prior to this patients were swabbed if they had symptoms suggestive of COVID or had risk factors for exposure. Nasopharyngeal swabs were performed by nursing or medical staff and sent for polymerase chain reaction testing.

Relevant concurrent illnesses were recorded, and classed as medical, gynaecological or pregnancy. Medical concurrent illnesses were those requiring therapy only available in a hospital setting. Gynaecological illnesses were acute gynaecological issues diagnosed on imaging or laparoscopy that could have accounted for the patients presenting symptoms. In all cases, pregnancy was known prior to presentation and confirmed on serum βHCG or ultrasound.

Complications recorded included conversion to open procedure, reoperation, intensive care admission, representation within 30 days and death. Post operative complications were also recorded in the following categories; medical, bleeding, collection and surgical site infection. Medical complications were defined as acute medical issues that arose in the postoperative period that required therapy and/or subspecialty consultation, such as new onset atrial fibrillation, labile blood glucose or pneumonia. Bleeding was defined as postoperative intra-abdominal or wound bleeding resulting in a haemoglobin drop of > 15 g/L or blood transfusion. Patients were recorded to have a collection if they had radiological evidence of a collection at the site of the appendicectomy in the postoperative period. Surgical site infections were defined as infections of operative incisions requiring oral or intravenous antibiotics, or wound packing.

Rates of complicated appendicitis were determined by intraoperative macroscopic findings and histopathology results. Patients were classified as having complicated appendicitis based on the “complex appendicitis” macroscopic and microscopic findings described by Bhangu et al. [20]; histopathological results were preferenced when there was a disagreement between macro- and microscopic findings. A ‘normal’ appendix was based only on histopathology.

### Statistical analysis

Statistical analysis was performed using STATA software version 16.1. Data was expressed as means and standard deviations for continuous variables, and as proportions or frequencies for categorical variables. Associations between categorical variables were assessed with either Chi Square or Fisher’s Exact tests. Based on whether the data had a normal distribution, associations between continuous variables were assessed using Unpaired T Test or Mann Whitney U tests. p values were deemed significant if p ≤ 0.05.

## Results

### Patient demographics

A total of 1556 patients were included in the operative arm of the study; 723 in the COVID group and 833 in the control group. The patient demographics are demonstrated in Table 1. In the COVID group, 1.66% had a positive COVID swab. The groups were comparable for age, gender and body mass index (BMI), as well as insurance status, concurrent diagnosis on presentation and number of previous abdominal surgeries. There was no significant difference in patient reported duration of symptoms on presentation.

**Table 1:** Patient demographics and presenting characteristics—operative cohort

Variable		Operatively managed appendicitis Pre-COVID pandemic (n = 833)	Operatively managed appendicitis during COVID pandemic (n = 723)	p-value
Age at operation, mean (range)		38.1 (18–71)	38.8 (18–93)	0.42
Gender	Female	400 (49.1%)	326 (46.2%)	0.24
	Male	415 (50.9%)	380 (53.8%)	
COVID result	No swab		242 (33.5%)	
	Negative		469 (64.9%)	
	Positive		12 (1.7)	
Symptomatic COVID	No		9 (1.2%)	
	Yes		3 (0.4%)	
BMI, mean (range)		27.8 (16.6–63.9)	28.5 (16.9–76.1)	0.17
Concurrent diagnosis on presentation	None	806 (97.0%)	691 (96.6%)	0.88
	Pregnant	8 (1%)	8 (1.1%)	
	Gynaecological	6 (0.6%)	3 (0.4%)	
	Medical	12 (1.4%)	13 (1.8%)	
Duration of abdominal pain (days), mean (range)		2.3 (1–30)	2.2 (1–28)	0.42
Number of previous abdominal operations, mean (SD)		0.3 (0.6)	0.3 (1.0)	0.22
Insurance status	Private	246 (29.7%)	203 (28.1%)	0.49
	Uninsured	586 (70.3%)	520 (71.9%)	

*BMI* body mass index, *SD* standard deviation

Fifty-two patients were included in the conservative arm of the study; 29 in the pandemic group and 23 in the control group. The demographics and characteristics at presentation are outlined in Table 2. There were two COVID positive patients managed conservatively during the pandemic. Patients that were managed conservatively were comparable for age, gender, BMI and insurance. Prior to the pandemic, 4 patients were managed conservatively due to patient choice, 16 due to a phlegmon, and 3 due to comorbidities. During the pandemic, 2 were managed conservatively due to patient choice, 20 due to a phlegmon, and 7 due to comorbidities.

**Table 2:** Patient demographics and characteristics at presentation—conservatively managed cohort

Variable		Conservatively managed appendicitis Pre-COVID pandemic (n = 23)	Conservatively managed appendicitis during COVID pandemic (n = 29)	p-value
Age, mean (range)		51.0 (24–76)	48.7 (25–82)	0.66
Gender	Female	13 (57%)	16 (55%)	0.92
	Male	10 (43%)	13 (45%)	
COVID result	No swab		0 (0%)	
	Negative		27 (93%)	
	Positive		2 (7)^†^	
BMI, mean (range)		27.0 (16.8–40.1)	27.2 (18–59.1)	0.92
Duration of symptoms (days), median (range)		7.0 (2–21)	5.0 (1–14)	0.0.51
Insurance status	Private	8 (35%)	7 (24%)	0.4
	Uninsured	15 (65%)	22 (76%)	

^†^Both asymptomatic

*BMI* body mass index

### Operative management of appendicitis

The outcomes of patients managed operatively in this study are outlined in Table 3. Patients presenting during COVID were significantly more likely to be investigated with CT scan, rather than being managed based on clinical presentation (p ≤ 0.001). In the COVID cohort patients were significantly less likely to have a normal appendix on histopathology (p = 0.001), reflecting a significant reduction in the negative appendicectomy rate.

**Table 3:** Operative cohort outcomes

Variable		Operatively managed appendicitis Pre-COVID pandemic (n = 833)	Operatively managed appendicitis during COVID pandemic (n = 723)	Odds ratio (95% confidence interval)	p-value
Length of stay (days), mean (SD)		3.1 (2.6)	3.1 (3.0)		0.86
Imaging	No imaging	175 (21.0%)	102 (14.1%)	1.6192 (1.2390–2.1161)	< 0.001
	CT	521 (62.5%)	518 (71.6%)		
	US	137 (16.4%)	102 (14.1%)		
	MRI	0 (0.0%)	1 (0.1%)		
Operation performed	Appendicectomy	764 (91.7%)	653 (90.3%)	0.8012 (0.5652–1.1358)	0.005
	Caecectomy	15 (1.8%)	35 (4.8%)		
	Laparoscopy	22 (2.6%)	11 (1.5%)		
	Laparotomy	16 (1.9%)	9 (1.2%)		
	Right Hemicolectomy	16 (1.9%)	15 (2.1%)		
Laparoscopic converted to open	No	804 (96.5%)	700 (96.8%)		0.74
	Yes	29 (3.5%)	23 (3.2%)		
ASA Score	1	383 (46.0%)	267 (37.0%)		0.001
	2	358 (43.0%)	349 (48.3%)		
	3	82 (9.9%)	96 (13.3%)		
	4	7 (0.8%)	10 (1.4%)		
Time from triage to operation (hours), median IQR		12.3 (6.4, 18.3)	14.1 (7.5, 19.1)		0.017
Complicated appendicitis	No	659 (79.1%)	540 (74.7%)	1.2835 (1.0130–1.6262)	0.039
	Yes	174 (20.9%)	183 (25.3%)		
Normal appendix	No	749 (89.9%)	682 (94.3%)	0.5360 (0.3638–0.7898)	0.001
	Yes	84 (10.1%)	41 (5.7%)		
Admission WBC (× 10^9^/L), mean (SD)		12.7 (4.8)	12.6 (5.3)		0.67
Admission CRP (mg/L), mean (SD)		48.6 (70.1)	44.2 (64.9)		0.21
Reoperation	No	842 (98.9%)	715 (99.0%)		0.83
	Yes	9 (1.1%)	7 (1.0%)		
Drain	No	680 (81.6%)	552 (76.3%)	1.3768 (1.0773–1.7596)	0.01
	Yes	153 (18.4%)	171 (23.7%)		
Post operative complication	No	807 (96.9%)	690 (95.4%)		0.14
	Yes	26 (3.1%)	33 (4.6%)		
ICU Admission	No	818 (98.2%)	707 (97.8%)		0.85
	Operation related	10 (1.2%)	10 (1.4%)		
	Other	5 (0.6%)	6 (0.8%)		
Readmission within 30 days	No	796 (95.6%)	692 (95.7%)		0.58
	Operation related	18 (2.2%)	19 (2.6%)		
	Other	19 (2.3%)	12 (1.7%)		
Death		0	0		

*SD* standard deviation, *CT* computed tomography, *US* ultrasound, *MRI* magnetic resonance imaging, *ASA* American Society of Anaesthesiologists, *IQR* interquartile range, *WBC* white blood cell count, *CRP* C-reactive protein, *ICU* Intensive Care Unit

Patients during the pandemic were also more likely to present with complicated appendicitis (p = 0.03), require caecectomy (p = 0.005) and had higher American Society of Anaesthesiologists (ASA) scores (p = 0.001). Right hemicolectomies were performed in 16 patients prior to the pandemic, five of which were due to suspicion for a malignancy macroscopically. Four of these patients were found to have a tumour on their histopathology. During the pandemic, 15 patients underwent a right hemicolectomy, however none were suspected to have a tumour intraoperatively, and this was confirmed by their histopathology. Drains were also more commonly used during the pandemic (p = 0.01). Despite the higher rates of complicated appendicitis, at presentation, there was no significant difference in the admission white blood cell count (p = 0.67) or C-reactive protein (p = 0.47).

Twelve patients (1.66%) presented with COVID and appendicitis, none of whom had symptomatic COVID. The patients aged between 21 and 73 years, with two of them being female, and 5 had private insurance. On presentation, patients had symptoms from 1 to 6 days, and nine were investigated with CT, one with US and two were diagnosed clinically. Mean length of stay was 2.34 days (range 1.1–4.9 days). All patients underwent a laparoscopic appendicectomy, except one whom required a right hemicolectomy for perforated appendicitis. All patients had uncomplicated appendicitis, and there were no reported postoperative complications, admissions to Intensive Care Unit (ICU), readmissions or deaths.

### Conservative management of appendicitis

The outcomes of patients managed conservatively in this study are outlined in Table 4. During the COVID pandemic, there was no significant difference in the length of stay, choice and duration of intravenous antibiotics, duration of oral antibiotics on discharge, radiological drainage of collections or admission inflammatory markers. In the cohort of patients managed conservatively during the pandemic, there was a significant reduction in the rates of complications (p = 0.033), readmissions (0.044) and interval appendicectomy (p = 0.0044).

**Table 4:** Conservatively managed patient outcomes

Variable	Conservatively managed appendicitis Pre-COVID pandemic (n = 23)	Conservatively managed appendicitis COVID pandemic (n = 29)	Odds ratio (95% confidence interval)	p-value
Length of stay (days), mean (SD)	6.4 (6.3)	6.0 (3.3)		0.76
Duration of IV antibiotics (days), mean (SD)	5.9 (5.3)	5.6 (3.5)		0.82
Duration of oral antibiotics on discharge (days), mean (SD)	9.4 (4.9)	6.9 (4.5)		0.069
IR drainage				
No	16 (70%)	20 (69%)		0.96
Yes	7 (30%)	9 (31%)		
Complication				
No	19 (83%)	29 (100%)	0.0734 (0.0037–1.4421)	0.033
Yes	4 (17%)			
Readmission				
No	15 (65%)	26 (90%)	0.2163 (0.0497–0.9421)	0.044
Yes	8 (35%)	3 (10%)		
Admission WBC (× 10^9^/L), mean (SD)	12.1 (4.5)	11.2 (3.7)		0.43
Admission CRP (mg/L), mean (SD)	85.6 (80.9)	120 (104.8)		0.2
Interval appendicectomy				
No	15 (65%)	26 (90%)	0.2163 (0.0497–0.9421)	0.044
Yes	8 (35%)	3 (10%)		

*SD* standard deviation, *IR* interventional radiology, *WBC* white blood cell count, *CRP* C-reactive protein

## Discussion

The SWSLHD services a population of more than 1 million people [21] with pockets of diversity and social disadvantage [11]. When compared to the NSW state average, the population serviced by the health district has a lower than average level of education attainment and weekly income [22]. The population is also more ethnically diverse than the NSW state average, with 45.2% of the population being born overseas (more than 10% higher than the NSW average) and 54% speaking a language other than English at home; more than double the NSW average [22]. The population experienced some of the highest case numbers, hospitalisations, ICU admissions and deaths during the pandemic, particularly in the Delta and Omicron waves. The district provides an insight into the impact of the pandemic on the delivery of emergency surgical care in a population reliant on the public health system.

This is the largest Australian multicentre study to examine the impact of COVID 19 pandemic during the peak of cases experienced during the Delta and Omicron waves. We identified a significant shift in the management of patients presenting with acute appendicitis. Firstly, there was an increase in the reliance on imaging to diagnose patients. We demonstrated a significant rise in the number of patients presenting with complicated appendicitis, and this potentially accounts for the greater number of caecectomies performed and use of intraoperatively inserted drains. Our study identified that patients presenting during the pandemic had a longer wait time from triage to operation. In our cohort, we also saw a reduction in the rates of negative appendicectomies. Patients managed conservatively during the pandemic, were less likely to experience a complication, be readmitted and undergo an interval appendicectomy.

This study identified there was a statistically significant reduction in clinical diagnosis of appendicitis during the pandemic; patients presenting prior to the pandemic were twice as likely to be managed based on their clinical presentation. Greater use of CT to diagnose appendicitis during the pandemic was also reported in the United Kingdom [23, 24], Ireland [25], Middle East [26] and Turkey [27]. These studies have reported greater utilisation of CT ranging from increases of 21–123%. We hypothesise that this may have occurred due to a number of reasons. Firstly, by ensuring patients had radiological evidence of appendicitis, surgeons would have been able to justify a hospital admission and use of hospital resources. Alternatively, there may have been a higher rate of outpatient imaging prior to presenting to hospital. Unfortunately, the rates of inpatient or outpatient scans were not recorded at time of data collection, and would be an important future project. Thirdly, patients may have had a higher threshold to present to hospital, due to fear of contracting COVID; therefore resulting in fewer patients with self-limiting right iliac fossa pain presenting to the hospital.

The greater use of CT imaging during the pandemic is likely to explain the lower negative appendicectomy rate seen during the pandemic. Outside of the pandemic, a negative appendicectomy rate of 9.7% was reported in a retrospective study of 8206 patients [28], which is consistent with what is widely reported in the literature. In our cohort, the negative appendicectomy rate nearly halved during the pandemic to 5.7%, whilst the rate of CT prior to operation increased by approximately 10%. Studies during the pandemic also noted the same significant reduction in negative appendicectomy, and have reported a rate as low as 0% [23, 25, 26, 29]. Their studies also identified higher rates of CT use. Increased use of CT has also been demonstrated in a number of populations outside of the pandemic to be associated with a lower negative appendicectomy rate [30, 31].

The average cost of performing an appendicectomy in Australian metropolitan and regional hospitals in 2011–12 was $6300 Australian Dollars (AUD) [32]. In contrast a CT scan of the abdomen and pelvis has a government rebate of $499.50 AUD [33]. The management of appendicitis during the pandemic has highlighted that using CT to investigate patients may reduce health expenditure and avoid unnecessary operations; outcomes that are desirable to patients, clinicians and policy makers. Greater use of CT imaging is not without risk; the additional radiation exposure and potential risks to patients must also be considered.

During the pandemic we identified a statistically significant increase in the rates of complicated appendicitis. A rise in the number of patients presenting with complicated appendicitis has also been demonstrated in other studies [33, 34], with reported increased relative risk of 1.55 in a meta-analysis of 3559 patients [36]. We believe the significant increase in complicated appendicitis can account for the significant increase in rates of caecectomy and intraoperative insertion of drains. Other studies that recorded rates of caecectomy during the pandemic did not identify a significant increase [37–39]. Only one of these studies identified a significant increase in the rates of complicated appendicitis during the pandemic, and all were conducted in Korea and therefore examined a different population and health system.

It has been postulated that patient fear of presenting to hospital during the pandemic may have been responsible for delayed presentation and thus resulting in increased rates of complicated appendicitis, however, patient reported duration of symptoms on presentation did not significantly differ between the pandemic and control group. This finding has been reflected in a Turkish study of 377 patients, which found there was no statistical difference in patient reported duration of symptoms, prior to and during the pandemic [40]. The accuracy of patient reported duration of symptoms can potentially vary between patients, as they are a subjective measure, and therefore may not be an accurate predictor of complicated appendicitis. Other studies that have identified an increase in complicated appendicitis, have identified a delay in presentation [34, 41]. However, the subjective nature of these measures does not exclude the possibility that patients did in fact delay their presentation during the pandemic due to fear of exposure to COVID in hospital. This could be examined by a study of patient attitudes.

Another postulated cause of the increased complicated appendicetomy rates may have been a delay to performing operation due to increased loads on the emergency department, imaging and medical staff during the pandemic. Adults presenting during the COVID-19 pandemic had a significantly longer time from triage to operation, however the means differed by two hours, and therefore is unlikely to explain the difference in rates of complicated appendicitis pre- and during the COVID-19 pandemic.

Patients undergoing operative management for appendicitis during the pandemic were assessed to have poorer overall health, as determined by their significantly higher ASA scores. This was not identified in other studies, which all reported no significant difference between the pandemic and control cohort ASA scores [37, 38, 42]. There are a number of explanations why our findings differed from other studies; the studies were conducted in different countries with differing health care systems, and the ASA is a subjective measure. Concurrent COVID infection is unlikely to account for the higher ASA scores in our cohort, as COVID positive patients accounted for only 1.66% of the pandemic cohort in the study. The pandemic resulted in variable changes in the health behaviours of some Australians, and the way in which the community accessed health care, which could explain this finding [43]. However, it is beyond the scope of this study to examine for variables that could account for high ASA scores.

Our study also examined trends for conservatively managed appendicitis during the pandemic. During the pandemic there were reduced rates of readmissions and complications, and a shorter duration of oral antibiotics on discharge. Whilst the study has identified some statistically significant trends in the conservative management of appendicitis, there is a high risk of type I error due to the low incidence of these outcomes of interest, and therefore it is not possible to comment on the differences observed, and a larger study is required.

## Conclusion

The COVID 19 pandemic resulted in a shift in the management of acute appendicitis; there was a significant increase in the reliance on imaging to diagnose patients, which likely accounts for the observed reduction in the negative appendicectomy rate. Patients presented with higher rates of complicated appendicitis, and there were more caecectomies performed.

## Data Availability

The datasets generated and analysed during the current study are not publicly available as this was the conditions on which ethics approval was provided. However, the dataset could be provided on reasonable request from the corresponding author and with ethics committee approval.

## Abbreviations

COVID-19/COVID: Coronavirus disease 2019
SARS-CoV2: Severe acute respiratory syndrome coronavirus 2
SWSLHD: South Western Sydney Local Health District
NSW: New South Wales
CT: Computerised tomography
US: Ultrasound
BMI: Body mass index
ASA: American Society of Anaesthesiologists
AUD: Australian Dollars
